# The Pathogenicity of *Pseudomonas syringae* MB03 against *Caenorhabditis elegans* and the Transcriptional Response of Nematicidal Genes upon Different Nutritional Conditions

**DOI:** 10.3389/fmicb.2016.00805

**Published:** 2016-05-30

**Authors:** Muhammad Ali, Yu Sun, Li Xie, Huafu Yu, Anum Bashir, Lin Li

**Affiliations:** ^1^State Key Laboratory of Agricultural Microbiology, Huazhong Agricultural UniversityWuhan, China; ^2^Biotechnology Program, Department of Environmental Sciences, COMSATS Institute of Information TechnologyAbbottabad, Pakistan

**Keywords:** *Pseudomonas syringae* MB03, *Caenorhabditis elegans*, pathogenicity, transcriptional response, gut colonization

## Abstract

Different species of the *Pseudomonas* genus have been reported for their pathogenic potential against animal cells. However, the pathogenicity of *Pseudomonas syringae* against *Caenorhabditis elegans* has never been reported. In this study, the interaction of *P. syringae* MB03 with *C. elegans* was studied. Different bioassays such as killing assay, lawn leaving assay, food preference assay, L4 growth assay and newly developed “secretion assay” were performed to evaluate the pathogenic potential of *P. syringae* on different growth media. The results of the killing assay showed that *P. syringae* MB03 was able to kill *C. elegans* under specific conditions, as the interaction between the host and the pathogen varied from non-pathogenic (assay on NGM medium) to pathogenic (assay on PG medium). The lawn leaving assay and the food preference assay illustrated that *C. elegans* identified *P. syringae* MB03 as a pathogen when assays were performed on PG medium. Green fluorescent protein was used as the reporter protein to study gut colonization by *P. syringae* MB03. Our results suggested that MB03 has the ability to colonize the gut of *C. elegans.* Furthermore, to probe the role of selected virulence determinants, qRT-PCR was used. The genes for pyoverdine, *phoQ/phoP, phoR/phoB*, and flagella were up regulated during the interaction of *P. syringae* MB03 and *C. elegans* on PG medium. Other than these, the genes for some proteases, such as *pepP*, *clpA*, and *clpS*, were also up regulated. On the other hand, *kdpD* and *kdpB* were down regulated more than threefold in the NGM – *C. elegans* interaction model. The deletion of the *kdpD* and *kdpE* genes altered the pathogenicity of the bacterial strain against *C. elegans*. Overall, our results suggested that the killing of *C. elegans* by *P. syringae* requires a prolonged interaction between the host and pathogen in an agar-based assay. Moreover, it seemed that some toxic metabolites were secreted by the bacterial strain that were sensed by *C. elegans*. Previously, it was believed that *P. syringae* could not damage animal cells. However, this study provides evidence of the pathogenic behavior of *P. syringae* against *C. elegans*.

## Introduction

The bacterial genus *Pseudomonas* consists of many important species, ranging from plant, animal, and human pathogens to soil inhabitants. Of the various pathogenic species within this genus, the human opportunistic pathogen *Pseudomonas aeruginosa* and the plant-pathogenic *Pseudomonas syringae* have gained attention over other species (Mahajan-Miklos et al., 1999; Lindeberg et al., 2012). Numerous studies have been conducted to elucidate the mechanism of *P. aeruginosa* against the model organism, *Caenorhabditis elegans* (Zaborin et al., 2009; Kirienko et al., 2013), which have unveiled the cumulative action of different virulence-related genes (Lee et al., 2006). To date, the various mechanisms by which *P. aeruginosa* strains kill *C. elegans*, such as red death, lethal paralysis, agar-based slow killing, agar-based fast killing, and liquid killing, have been reported (Darby et al., 1999; Zaborin et al., 2009; Utari and Quax, 2013), and a variety of virulence factors found in this bacterium are common to many other *Pseudomonas* species (Dubern et al., 2015). However, the role of these factors in different infection models needs to be tested, as the contribution of a certain gene to its pathogenicity may depend upon the infection model.

It has been known that the virulence factors of one bacterial strain may not have any role in the pathogenicity of another strain (Lee et al., 2006). Even in some cases, the same gene can affect bacterial virulence in different manners. For instance, deletion of *kdpD–kdpE* genes resulted in enhanced virulence of *Mycobacterium tuberculosis* whereas isogenic mutant of *kdpD* gene decreased colonization ability of *Salmonella typhimurium* (Parish et al., 2003; Alegado et al., 2011). Moreover, it has been reported that the host–microbe interaction is shaped by the model host and environmental conditions (Dubern et al., 2015). Previously, it was reported that a change in the composition of growth medium results in altered pathogenicity (Mahajan-Miklos et al., 1999). Even a change in the physical form of the pathogenicity assay (solid killing assay vs. liquid killing assay) also affected the rate of nematode killing (Kirienko et al., 2013). These findings strengthen the idea that the study of the transcriptional response of a set of genes under virulence enhancing and virulence repressing conditions may provide clues for the mechanism of bacterial virulence.

Several other dominant *Pseudomonas* species, such as *P. fluorescens*, *P. putida*, and *P. syringae*, were generally perceived as non-pathogenic to animal cells. However, some recent investigations have revealed that *P. putida* and *P. chlororaphis* possess the potential to damage animal tissues (Fernandez et al., 2015; Nandi et al., 2015). In the case of *P. syringae*, the core genome was determined from phylogenetically diverse strains, and the analysis revealed approximately 2,500 common genes among the core genome (3,397 genes) of *P. syringae*, *P. fluorescens*, and *P. putida* (Baltrus et al., 2011). However, the interaction between *P. syringae* and *C. elegans* was found to be non-pathogenic when the assay was conducted on NGM agar. Interestingly, variable behavior of *P. fluorescens* was observed against *C. elegans*, as the worms were only repelled on NGM agar, whereas the interaction became lethal on BHI agar medium (Burlinson et al., 2013). These findings suggest that an adequate infection model should be set up to study the pathogenicity of certain *P. syringae* strains.

Bacterial two component systems, such as *kdpD*/*kdpE, gacA*/*gacS, phoQ*/*phoP, and phoB*/*phoR* have been demonstrated for their impact on virulence. Previous studies have revealed that the *kdpD*/*kdpE* system is responsible for potassium homeostasis and regulating the virulence of different pathogenic bacteria, such as enterohemorrhagic *Escherichia coli*, *Salmonella typhimurium*, *Mycobacterium tuberculosis*, and *Yersinia pestis* (Parish et al., 2003; O’Loughlin et al., 2010; Alegado et al., 2011; Njoroge et al., 2012). In *Staphylococcus aureus* and *Mycobacterium avium*, the *kdpD* and/or *kdpE* genes were differentially expressed during the host–pathogen interaction (Hou et al., 2002; Voyich et al., 2005). In the current study, we investigated the potential of a plant-pathogenic *P. syringae* strain with high ice-nucleating activity, *P. syringae* MB03 (Li et al., 2012), to harm the model organism *C. elegans*. We performed multiple bioassays including killing assay, lawn leaving assay, food preference assay, L4 growth assay and newly developed “secretion assay” to evaluate the pathogenic potential of *P. syringae* on different growth media. We examined the transcriptional response of homologs of selected nematicidal genes of *P. aeruginosa* PA14 and *P. aeruginosa* PAO1. We also analyzed the role of the genes *kdpD* and *kdpE* in the pathogenicity of *P. syringae* MB03 against *C. elegans*. All of these experiments aim at providing the first evidence that the conventional plant-pathogenic *P. syringae* has the potential to kill *C. elegans*.

## Materials and Methods

### Bacterial and *C. elegans* Strains, Media, Genes, Plasmids, and Culture Conditions

The bacterial strains and plasmids used in this study are listed in **Table 1**. *P. syringae* wild-type strain MB03 (CCTCC No. M2014114, China Center for Type Culture Collection) was used for the infection experiments against *C. elegans*. The *kdpD*- and *kdpE*-disrupted mutant strains of *P. syringae* MB03, called *P. syringae* MB03Δ*kdpD* and *P. syringae* MB03Δ*kdpE*, respectively, were constructed to study the role of the two component system of *kdpD*/*kdpE* during the infection. The transformed *P. syringae* MB03 harboring the recombinant p519ngfp was named as *P. syringae* MB519 and it was used as an indicator through the expression of green fluorescent protein (GFP). The synchronized fourth-stage (L4) larvae of *C. elegans* wild-type strain N2 (Bristol) were used for the bioassays. Assays were performed using four different media, including PG (Tan et al., 1999a), BHI (Powell and Ausubel, 2008), King’s B (King et al., 1954), and NGM (Bischof et al., 2006). All of the selected media have different nutrient compositions, and these media were selected to perform the assay because these were the host favoring condition (NGM), the pathogenicity favoring condition (PG), the siderophore (secondary metabolite) secretion medium (King’s B), or the pathogen growth supporting medium (BHI).

**Table 1:** Bacterial strains and plasmids used in this study.

Strains and plasmids	Phenotypes	Reference
***Pseudomonas syringae strains***		
MB03	Wild-type strain with highly ice-nucleating activity	Li et al., 2012
MB03Δ*kdpD*	*kdpD* disrupted mutant of MB03	This study
MB03Δ*kdpE*	*kdpE* disrupted mutant of MB03	This study
MB519	Transformed MB03 strain containing p519ngfp for expression of green florescent protein, Kanamycin^+^	This study
DK519	Transformed Δ*kdpD* strain harboring p519ngfp plasmid to express green florescent protein, Kanamycin^+^	This study
EK519	Transformed Δ*kdpE* strain harboring p519ngfp plasmid to express green florescent protein, Kanamycin^+^	This study
***P. aeruginosa strains***		
PAF	Wild-type strain isolated from agricultural soil	This study
***Escherichia coli strains***		
WM3064	Diaminopimelic acid (DAP) auxotroph strain used for conjugation and maintaining pDS3.0 vector, Gentamicin^-^, Sucrose^+^	Saltikov and Newman, 2003
WMDK	Transformed *E. coli* WM3064 strain harboring suicide plasmid pDSDK for *kdpD* knockout, Gentamicin^+^, Sucrose^-^	This study
WMEK	Transformed *E. coli* WM3064 strain harboring suicide plasmid pDSEK for *kdpE* knockout, Gentamicin^+^, Sucrose^-^	This study
OP50	The food source used for *C. elegans* N2	
**Plasmids**		
pDS3.0	Suicide plasmid used for gene knockout, Gentamicin^+^, Sucrose^-^	Gao et al., 2006
pDSDK	A pDS3.0 derivative plasmid containing upstream and downstream fragments for *kdpD* knockout, Gentamicin^+^, Sucrose^-^	This study
pDSEK	A pDS3.0 derivative plasmid containing upstream and downstream fragments for *kdpE* knockout, Gentamicin^+^, Sucrose^-^	This study
p519ngfp	Green fluorescent protein expressing vector, Kanamycin^+^	Matthysse et al., 1996

### Bioassays

Different bioassays were performed to evaluate the pathogenicity of *P. syringae* MB03 against *C. elegans.* These assays include: (1) killing assay; (2) lawn leaving assay; (3) food preference assay; (4) secretion assay; and (5) growth assay. The detailed procedure of each assay is provided in the following section.

#### Killing Assay

*Pseudomonas syringae* MB03 was grown in PG, BHI, King’s B, and NGM media overnight, and 15 μl of the cell suspension with an OD_600_ of 0.5 was evenly spread on each respective medium. Then, 40–50 L4 worms were placed on the bacterial lawn, and the survival rate was determined after the specified time intervals. The bioassays against *C. elegans* L4 worms were performed according to previously described methods (Bischof et al., 2006; Powell and Ausubel, 2008). Lethality was evaluated at specified time intervals by probing the larvae with a dissecting needle under a stereo microscope. Worms that did not respond to touch were considered dead.

#### Lawn Leaving Assay

To perform lawn leaving assay, *P. syringae* MB03 was grown in the respective medium for overnight. Bacterial lawn was prepared by carefully spotting the bacterial suspension on plates and then incubating them at 28°C for 24 h. Approximately, 40–50 L4 worms were placed at the center of the bacterial lawn and plates were incubated at 25°C. The number of worms within and outside of the bacterial lawn was determined every 5 h.

#### Food Preference Assay

To determine the food preference of *C. elegans*, *E. coli* OP50 and the test strains (*P. syringae* MB03 and mutants) were grown overnight. Lawns of the test strain and the *E. coli* OP50 strains were made by spotting the bacterial suspension on all growth media. The resulting plates were incubated at 28°C for 24 h. *C. elegans* L4 worms were placed at the center of the plate at an equal distance to both bacterial lawns and plates were incubated at 25°C. The number of worms present in both lawns was determined after 10 h.

#### Secretion Assay

A secretion assay was designed to investigate the role of bacterial toxic secretions of *P. syringae* MB03 and its mutants into the medium. Test bacterial strains were grown on 0.22 μm filter paper for 24 h. After 24 h, the filter papers were removed from the agar, and *E. coli* OP50 was grown on the plates in such a way that two separate bacterial lawns were prepared, one at the place of the filter paper of the test strain, designated as the “test lawn,” and other at the opposite end to test strain lawn designated as the “OP50 lawn.” L4 worms were placed in the center of the plate (at an approximately equal distance from both lawns), and the number of worms on both lawns was determined after 15 h. The choice index (*C*_i_) was determined using the following formula:

(1) $$C_{i}=\frac{N_{ts}-N_{o}}{N_{a}}\,\;\;\;\;\;\;\;\;\;\;\;\;\;\;\;\;\;\;\;\;\;\;\;\;\;\;\;\;\;\;\;\;\;\;\;\;\;\;\;\;$$

where, *N*_ts_ denotes the number of worms on the test lawn, *N*_o_ denotes the number of worms on the OP50 lawn, and *N*_a_ denotes the total number of worms.

#### Growth Size Assay

Bacterial strains were grown overnight to prepare inoculums. Fifteen microliters of the overnight culture was spread over growth media to prepare bacterial lawn. *C. elegans* L4 synchronized worms were placed on the lawn and plates were incubated at 25°C. Images were taken at regular intervals in square pixels. To determine relative size of worms, the mean area of the worms fed on *P. syringae* MB03 was normalized to the mean area of worms grown over *E. coli* OP50.

### Real Time Quantitative Polymerase Chain Reaction (RT-qPCR)

*Pseudomonas syringae* MB03 was grown on PG, BHI, King’s B, and NGM media for 24 h at 28°C to prepare bacterial lawns. Synchronized L4 worms suspended in M9 buffer were transferred to the bacterial lawns, and the plates were incubated at 25°C. For control samples, only M9 buffer was added to the bacterial plates. Cells were collected after 24 h, and the RNA was extracted according to a previously reported method (Schmittgen and Livak, 2008). The cDNA was synthesized using the cDNA synthesis SuperMix (TransGen Biotech) with random primers. RT-qPCR was performed using a previously described method (Wang et al., 2011) to measure the mRNA levels of the selected potential nematicidal genes of *P. syringae* MB03. The reactions were performed in triplicate. The 16S rRNA gene and the *recA* gene were PCR amplified using the primer pairs 16S-F/16S-R and recA-F/recA-R, respectively, for use as internal controls (Supplementary Table S1). The comparative cycle threshold method (2^-ΔΔ^*^C^*^T^ method) was used to analyze the mRNA levels (Livak and Schmittgen, 2001).

The primers used for the RT-qPCR analysis of the potential nematicidal genes of *P. syringae* MB03 are listed in Supplementary Table S1. These genes were selected by the alignment analysis of the nematicidal genes identified from *P. aeruginosa* PAO1 and *P. aeruginosa* PA14 (Feinbaum et al., 2012; Dubern et al., 2015), which included the homolog genes for pyoverdine, flagella, type IV pilli, sigma factors, alginate, some two component system genes, such as *gacA*/*gacS*, *phoP*/*phoQ*, *phoB*/*phoR*, *kdpD*/*kdpE*, and other miscellaneous genes.

### Markerless Knockout of *kdpD* and *kdpE* Genes

Markerless mutants of *kdpD* and *kdpE* genes were constructed by SOE PCR. For the *kdpD* deletion, 1,003 bp upstream and 1,584 bp downstream fragments were amplified from the genomic DNA of *P. syringae* MB03 using the primers D5O-F/D5I-R and D3I-F/D3O-R, respectively. The PCR-amplified products were gel-purified and used in a second round of PCR to generate a fused fragment using D5O-F/D3O-R primers. In the construction of the markerless mutants, antibiotic resistance gene was not fused within the upstream and downstream fragments of the gene of interest. The second round of PCR resulted in a 2,587 bp fragment that was inserted into the pMD18-T vector (Takara) for sequence confirmation and cloning into *E. coli* DH5α. Furthermore, this 2,587 bp fused fragment was inserted into the pDS3.0 suicide vector (Gao et al., 2006) using a *Sac*I restriction site. The newly constructed vector, pDSDK, which contained the fused fragment for the *kdpD* knockout, was transferred into *E. coli* WM3064 (strain designated as *E. coli* WMDK). Subsequently, the suicide vector pDSDK was transferred into *P. syringae* MB03 (recipient strain) from *E. coli* WMDK (donor strain) by conjugation. Briefly, the donor and recipient strains were grown to mid log phase, and the cultures were mixed to a 3:1 ratio (donor:recipient). The mixed culture of the mating strains was harvested by centrifugation and poured onto the surface of LB agar (Hmelo et al., 2015). After an overnight incubation, the bacterial lawn was scratched and spread on LB^Gm+,DAP- (diaminopimelic acid)^ agar for the isolation of the merodiploid *P. syringae* strain. *E. coli* WMDK is a DAP auxotroph that failed to grow on LB medium^Gm+,DAP-^. Thus, the merodiploid *P. syringae* strain was isolated from the conjugation mixture (*P. syringae* MB03 and *E. coli* WMDK mixture). *P. syringae* mutants having the first homologous recombination (single cross over between the pDSDK plasmid and the chromosome of MB03) were screened for sucrose-negative and gentamicin-positive phenotypes. For the screening of the second homologous recombination mutants, a single cross over mutant was grown in LB medium, and the bacterial colonies were screened for sucrose-positive and gentamicin-negative phenotypes. To confirm gene knockout, the primers DKC-F/DKC-R were designed from the flanking regions of the further upstream and downstream regions that were not included in the gene knockout, and the amplified product was sequenced.

A similar procedure was used for the *kdpE* knockout, except the lengths of the upstream and downstream fragments were 1,053 bp and 973 bp, respectively. These fragments were ligated to generate a 2,026 bp fused fragment. Primer details and a schematic layout of the gene knock out methodology are presented in Supplementary Table S2 and **Supplementary Figure S1**, respectively.

### Gut Colonization

The plasmid p519ngfp expressing GFP (Matthysse et al., 1996) was introduced into *P. syringae* MB03 through electroporation (Dennis and Sokol, 1995). The resultant recombinant strain, *P. syringae* MB519, was grown on PG, BHI, King’s B, and NGM media to form bacterial lawns, and then, 80–100 age-synchronized L4 *C. elegans* worms were placed on the plates. At regular time intervals, the worms were repeatedly washed with M9 buffer (with 30 mM sodium azide as an anesthetic agent) and placed on a 2% agar pad on microscopic slides. The fraction of worms expressing GFP in its intestine was examined by fluorescence microscopy. Similar experiment was also performed on the mutant strains *P. syringae* MB03Δ*kdpD* and *P. syringae* MB03Δ*kdpE*. For this purpose, the plasmid p519ngfp was transferred into *P. syringae* MB03Δ*kdpD* and *P. syringae* MB03Δ*kdpE* to construct strains *P. syringae* DK519 and EK519, respectively, and the colonization experiments were performed in parallel following the procedures as described above.

## Results

### Host–Pathogen Interaction Varied from Non-pathogenic to Pathogenic with Different Media

**FIGURE 1:**
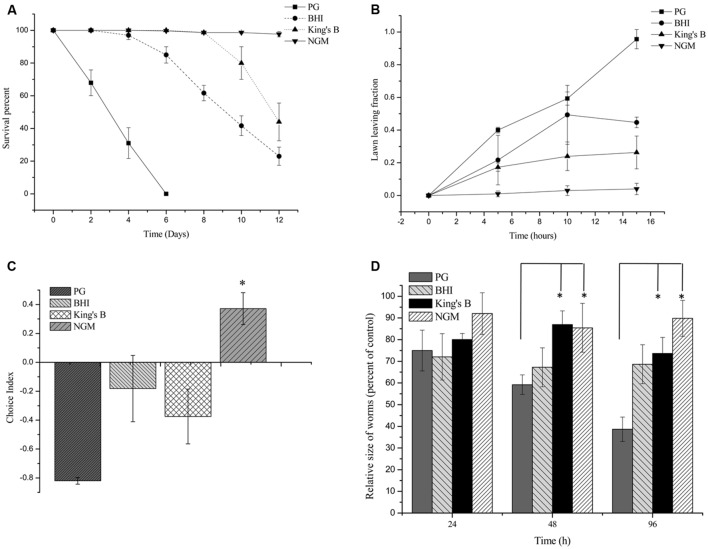
**Bioassays of *Pseudomonas syringae* MB03 against *Caenorhabditis elegans*. (A)** Killing of *C. elegans* by *P. syringae* MB03. Assay was conducted on four different media (PG, BHI, King’s B, and NGM). Fastest killing was observed on PG medium. **(B)** Shows lawn leaving behavior of *C. elegans*. *P. syringae* MB03 was grown on the PG, BHI, king’s B, and NGM media as bacterial lawn. L4 synchronized worms (40–50) were placed approximately at the center of bacterial lawn. Fraction of worms which was out of lawn was determined after regular time intervals. **(C)** Food preference assay shows choice index of *C. elegans* for *P. syringae* MB03 (-1 represents complete avoidance, 0 represents equal preference, and +1 represent complete preference). On PG medium, worms strictly avoided *P. syringae* MB03 whereas on NGM, worms did not avoid *P. syringae* MB03. **(D)** Growth of L4 worms on different media compared to control. L4 synchronized worms were placed on bacterial lawns and area of worms was determined. Worms grown on *E. coli* OP50 on same medium were used as control and normalized size of treated worm was determined by dividing treated worm area to worm grown on *E. coli* OP50. Statistical analysis was done by applying one-way ANOVA where * represents significant difference at *p*-value <0.05.

To investigate the pathogenic potential of *P. syringae* MB03 against *C. elegans*, different bioassays were performed using different growth media. Our results showed that *P. syringae* MB03 was more virulent on PG medium compared to the BHI and King’s B media. Interestingly, no death was observed on NGM medium (**Figure 1A**), verifying that the host–pathogen interaction changed from non-pathogenic to pathogenic with the change in growth medium. Previously, it was reported that *C. elegans* can sense different secreted metabolites of pathogenic bacteria and tries to avoid bacterial populations (Meisel et al., 2014). To investigate whether the worms were able to sense any toxic metabolites secreted by *P. syringae* MB03, we performed lawn leaving assays using four different media: PG, BHI, King’s B, and NGM. The *P. syringae* MB03 strain was grown on each medium for 24 h, and L4 synchronized worms (40–50) were placed on the center of the bacterial lawn. The fraction of the worms in and out of the lawn was determined after every 5 h. It was observed that a considerable fraction of *C. elegans* was out of the bacterial lawn of MB03 on PG medium. The lawn leaving behavior of the worms was obvious on PG medium, as approximately 0.9 of the total worms was out of the lawn after 15 h. Although a small fraction of the worms avoided the bacterial strain on King’s B and BHI media, the lawn leaving fraction was less than that found for the PG medium. On the other hand, the worms did not avoid the bacterial lawn grown on NGM medium (**Figure 1B**). Furthermore, we performed a food preference assay, as the food choice and aversive behavior of *C. elegans* have been previously verified (Zhang et al., 2005). Interestingly, the choice index of *C. elegans* for *P. syringae* MB03 varied from positive to negative by changing the growth media (**Figure 1C**), and *C. elegans* used *P. syringae* MB03 as a preferred food source on NGM medium. On PG, BHI, and King’s B media, *E. coli* OP50 was preferred over *P. syringae* MB03 by *C. elegans*. Moreover, a growth assay was performed in which L4 synchronized worms were grown on PG, BHI, King’s B, and NGM media. The size of the worms was determined every 24 h and normalized to the size of the worms grown on *E. coli* OP50 on the same medium. As shown in **Figure 1D**, a significant reduction of worm size (approximately 61%) was observed for the PG medium after 96 h of exposure, whereas no major reduction was observed on NGM medium. As MB03 was non-pathogenic to worms on NGM medium and serves as food source for worms, the ratio between worm area fed on MB03 and worm area fed on OP50 remained nearly constant.

### Newly Developed ‘Secretion Assay’

**FIGURE 2:**
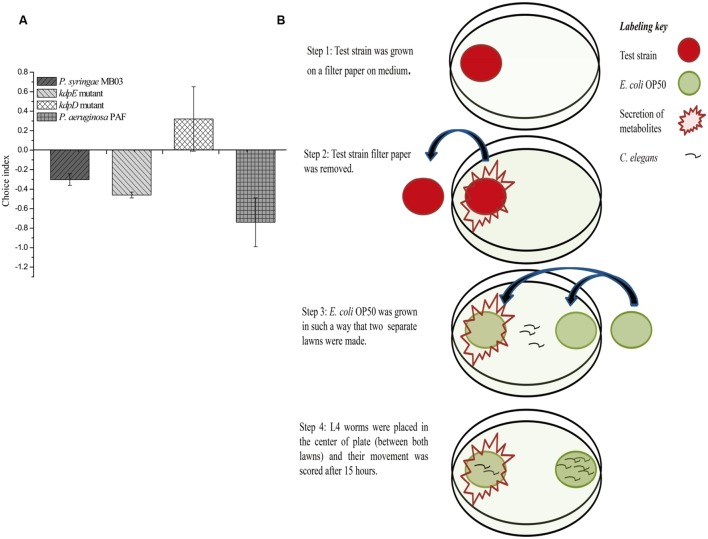
**Response of *C. elegans* to the secretion of *P. syringae* MB03, Δ*kdpD*, and Δ*kdpE*.** Newly developed secretion assay was performed to observe toxicity avoidance behavior of worms. Assay was performed on PG medium. **(A)** Shows choice index of worms for ‘test lawn’ (*E. coli* OP50 bacterial lawn made on secretion spot of test strains). *P. aeruginosa* PAF was used as control. **(B)** Schematic representation of secretion assay.

Previously, a filter assay was used to investigate the secretion of toxic compounds by pathogens (Darby et al., 1999). However, this assay can only be used if the secreted toxin is capable of killing worms, as the assay is based on killing. In the case of mild toxins (where the toxin alone is not sufficient to kill *C. elegans* or it kills it very slowly), conventional filter assay is not suitable. Secondly, conventional filter assay is time consuming for mild pathogens, as the killing of worms is required to measure the results. To overcome these problems, we developed a new “secretion assay” that is suitable to detect secretion metabolites with mild toxicity. Moreover, the assay is fast, as the results are based on the movement of worms, which can be easily analyzed after 10–15 h (**Figure 2**). In this assay, at first, test bacterial strain was allowed to secrete its metabolites, and after this, the test bacterial strain was removed. *E. coli* OP50 was grown on the exact spot of the secretion of the test bacterial strain (test lawn) and also opposite to that lawn (OP50 lawn). It was observed that worms tried to avoid the test lawn (*E. coli* OP50 lawn that was made over the secretion spot of the test strain), which suggests that *C. elegans* sensed the secretion of *P. syringae* MB03 as toxic.

### *Pseudomonas syringae* MB03 Colonizes the Gut of *C. elegans*

**FIGURE 3:**
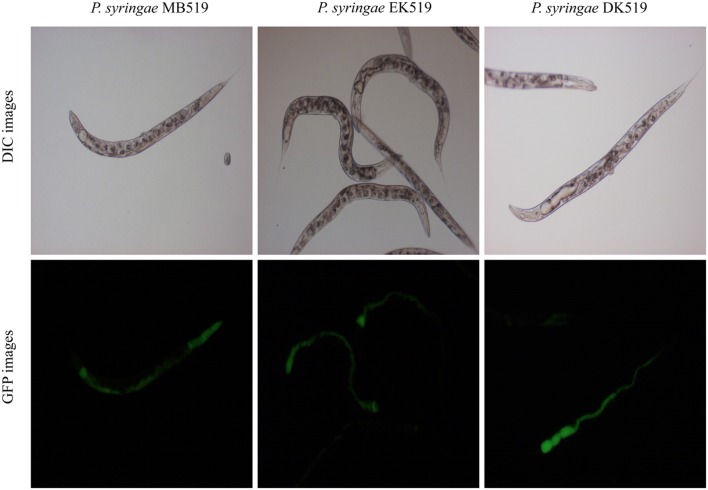
**Colonization of gut of *C. elegans* by *P. syringae* MB03, Δ*kdpD*, and Δ*kdpE*.** Worms were fed on bacterial strain harboring GFP expressing vector. At regular intervals worms were repeatedly washed with M9 buffer and examined under fluorescence microscope. Upper panel represents differential interference contrast image and lower panel represents florescence image of worm grown on *P. syringae* MB519 and *P. syringae* DK519 (Δ*kdpD*), *P. syringae* EK519 (Δ*kdpE*) on PG medium.

To investigate the colonization potential of *P. syringae* MB03, worms were fed on the bacterial strain expressing GFP. The assay was conducted on different media and at regular intervals, worms were analyzed for intestinal colonization by *P. syringae* MB03 (**Figure 3**). It was found that *P. syringae* MB03 had the ability to colonize the gut of *C. elegans*. Among the different media tested, PG supported colonization, whereas a negligible fraction of worms was colonized on the other media (BHI, King’s B, and NGM). Approximately, 47 ± 6% worms were colonized by *P. syringae* MB03 after 72 h exposure on PG medium. In the cases of King’s B and BHI media, fluorescence was undetectable. Similar to the other bioassay, *P. syringae* MB03 showed prominent gut colonizing potential on PG medium.

### Transcriptional Response of Potential Nematicidal Genes

**FIGURE 4:**
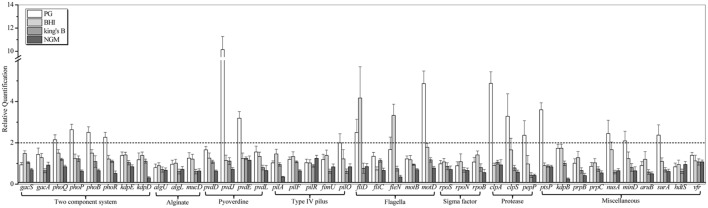
**Transcriptional variation of candidate virulence genes of *P. syringae* MB03 during host–pathogen interaction.** *P. syringae* MB03 was grown on PG, BHI, King’s B, and NGM media and then, L4 worms were added on bacterial lawns. After 24 h, total RNA was extracted for qRT-PCR analysis. *P. syringae* MB03 grown in the absence of worms was used as control sample for relative quantification of gene expression. Genes for *16S rRNA* and *recA* were used as indigenous control. Statistical analysis was done by applying one-way ANOVA.

To investigate the potential nematicidal genes, the genome of *P. syringae* MB03 was sequenced, and comparative genomics was applied to identify the homologs of the nematicidal genes compared to *P. aeruginosa* (Feinbaum et al., 2012; Dubern et al., 2015). *P. syringae* MB03 was grown on PG, BHI, King’s B, and NGM media for 24 h. After 24 h, L4 worms were added on one set of the plates, whereas another set (without *C. elegans*) was used as a control. Plates were incubated at 25°C, and the RNA was extracted after 24 h to study the regulation of selected genes (41 genes were selected) in the presence of the worms. In general, the up regulation of genes (17) was observed on PG medium, whereas on NGM medium, the down regulation of genes (8) was prominent (**Figure 4**, **Table 2**). Among different two component systems, the genes for *phoQ*/*phoP* and *phoB*/*phoR* were up-regulated when the assay was conducted on PG medium. Interestingly, *phoR* was down regulated (approximately twofold) on NGM medium in the presence of *C. elegans*. In the case of secondary metabolites, we observed an up regulation of greater than twofold for the pyoverdine genes, including *pvdJ* and *pvdE*. Some other genes, including *clpA*, *clpS*, *ptsP*, *nusA*, *mind*, *surA*, and *pepP*, showed more than twofold up regulation on PG medium. Interestingly, *pepP* was down-regulated (more than twofold) on King’s B and NGM media. Moreover, it was the only gene that showed more than a twofold change (±) on King’s B medium. None of the selected genes showed an up regulation on NGM medium. The most prominent down regulation was observed for the genes *kdpD*, *kdpB*, *pilA*, and *fleN* on NGM medium. Among these genes, *kdpD* and *kdpB* are the components of the two component *kdpD*/*kdpE* system and the *kdpFABC* (respectively) operon, and these two systems together maintain cellular homeostasis.

**Table 2:** Transcriptional response of selected genes of *P. syringae* MB03 in the presence of *C. elegans* on different media.

Genes	Media			
	PG	NGM	King’s B	BHI
Up-regulated*	17			2
Down-regulated*		8	1	

*Number of genes showing more than twofold variation.

### Role of *kdpD*/*kdpE* Two Component System in Pathogenicity

**FIGURE 5:**
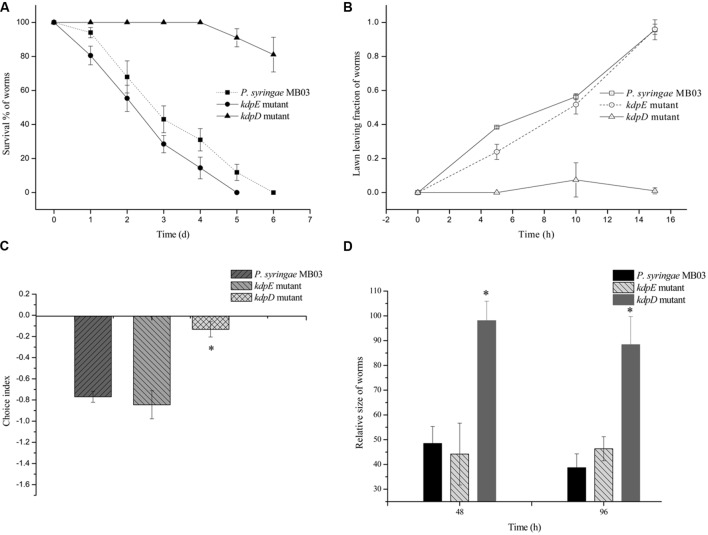
**Evaluation of pathogenicity of *P. syringae* MB03 and its mutants (Δ*kdpD* and Δ*kdpE*). (A)** For killing assay, all the test strains were grown over PG medium for 24 h and (40–50) L4 worms were added on bacterial lawn. Dead and live worms were determined after every 24 h. **(B)** Lawn leaving assay was performed on PG medium. Fraction of L4 worms out of the lawn was determined after every 5 h. **(C)** Food preference assay was conducted on PG medium. Fraction of worms on OP50 lawn and test strain lawn was determined after 15 h. **(D)** Growth assay was performed to determine effect of pathogenicity of test strain on the size of worms. Worms fed on *E. coli* OP50 were used as control to normalize size of treated worms. Statistical analysis was done by applying one-way ANOVA where * represents significant difference at *p*-value <0.05.

**FIGURE 6:**
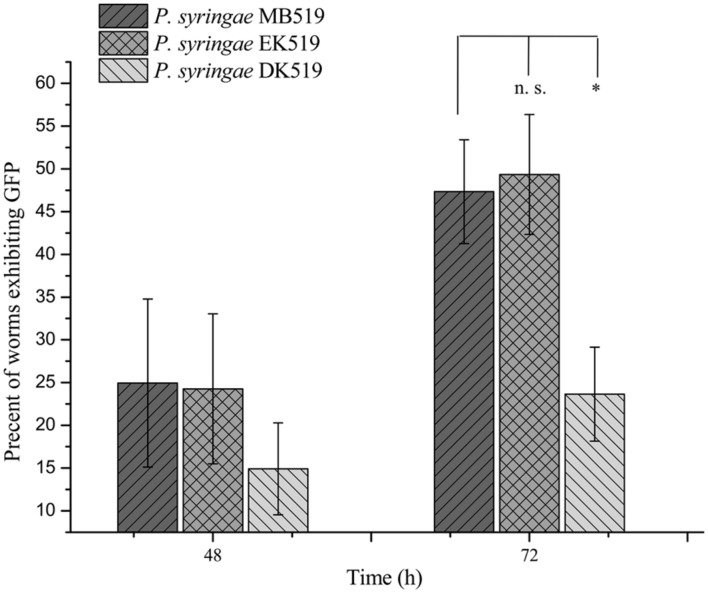
**Percent worms colonized by *P. syringae* MB519, *P. syringae* DK519, and *P. syringae* EK519.** GFP was expressed in *P. syringae* and its mutants. Strains were grown on PG medium and 80–100 L4 synchronized worms were fed on bacterial strains. Number of GFP expressing worms was determined under fluorescence microscope at regular intervals. Statistical analysis was done by applying one-way ANOVA where * represents significant difference at *p*-value <0.05 and ‘n.s.’ represent not significant results.

Previously, the *kdpD*/*kdpE* and *kdpB* genes have been reported for their role in bacterial virulence in different pathogenicity models (Alegado et al., 2011; Feinbaum et al., 2012; Njoroge et al., 2012; Dubern et al., 2015). However, these genes have never been investigated for their role in the pathogenicity of *P. syringae* against *C. elegans*. Based upon the experimental results of the transcriptional activity analysis of the *kdpD* and *kdpB* genes, we constructed two in-frame markerless mutants of the *kdpD* and *kdpE* genes. Different bioassays, including killing assay, lawn leaving assay, food preference assay, growth assay, and secretion assay, were conducted to evaluate the effect of the knockout on the pathogenicity of *P. syringae* MB03. The killing assay showed that the virulence of the *kdpD* mutant was noticeably attenuated, whereas the *kdpE* mutant showed enhanced virulence (**Figure 5A**). Although the *kdpD* mutant showed retarded growth, we performed a lawn leaving assay, food preference assay and secretion assay using this mutant. Our results showed that a very small fraction of worms avoided the lawn of *kdpD*, even after 15 h, whereas in the case of *P. syringae* MB03 and the *kdpE* mutant, a very high fraction of worms was out of the lawn at the same time point (**Figure 5B**). It seemed that the *kdpD* mutant was also deficient in toxin secretion. Similarly, a secretion assay was performed for *P. syringae* MB03, MB03*ΔkdpD*, and MB03*ΔkdpE*. Interestingly, we found a negative index for *P. syringae* MB03 and MB03*ΔkdpE*, whereas in the case of MB03*ΔkdpD*, a positive index was observed (**Figure 2A**). A positive index for MB03*ΔkdpD* showed the defect in its toxic secretion and a preference over *E. coli* OP50. Similarly, in the case of the food preference assay, the choice index was highly negative for *P. syringae* MB03 and MB03*ΔkdpE* compared to MB03*ΔkdpD* (**Figure 5C**). Worms highly preferred *E. coli* OP50 when the option was given among *E. coli* OP50 and *P. syringae* MB03 and MB03*ΔkdpE*. Similar results were observed in the growth assay where the worms grown on *P. syringae* MB03 and MB03*ΔkdpE* showed highly reduced size. However, no noteworthy reduction was observed in the case of MB03*ΔkdpD* (**Figure 5D**). Finally, we studied the effect of the gene knockout on the ability of the strains to colonize the gut. The plasmid p519ngfp was transferred to MB03*ΔkdpE* and MB03*ΔkdpD* to express GFP (**Supplementary Figure S2**). It was observed that MB03*ΔkdpD* was significantly retarded in its gut colonizing ability, whereas no significant difference was observed in the case of MB03*ΔkdpE* mutant (**Figures 3** and **6**). Collectively, our results demonstrated that the MB03*ΔkdpE* mutant appeared to be more virulent than the wild-type strain.

## Discussion

The pathogenicity of *P. syringae* against plants has been well documented. However, its interaction with a model animal, *C. elegans* for example, has not yet been investigated. The current study was designed to explore the pathogenic potential of *P. syringae* against *C. elegans*. Previously, *P. syringae* was reported as a harmless species to *C. elegans* and the bacterial lawns of strains DC3000 and B728a were grazed by worms (Burlinson et al., 2013). However, the study was conducted solely on NGM medium, a host favoring medium. In the present study, bioassays were conducted on different growth media, and our results showed a shift of the host–pathogen interaction from non-pathogenic (NGM) to pathogenic (PG medium). Collectively, the results suggested that *P. syringae* MB03 also secreted some metabolites that were toxic to *C. elegans*, as the worms avoided the bacterial lawn on PG medium. Previously, *P. aeruginosa* has been reported for secretion of toxic metabolites which were sensed by worms and subsequently led to the pathogen avoidance behavior in *C. elegans* (Meisel et al., 2014).

It has been reported that virulence factors are dependent upon the model host and environmental conditions (Dubern et al., 2015). It was assumed that key virulence determinants would show a varied transcriptional response in different host–pathogen interaction models. In general, the up regulation of potential virulence factors was observed on PG medium, whereas a prominent fraction of genes was down regulated when the host–pathogen interaction was studied on NGM medium (**Figure 4**, **Table 2**). Interestingly, no noticeable variation in the transcriptional response of most of the genes was observed on King’s B and BHI media.

Two component systems play an important role in sensing environmental changes and thus enable bacterial strains to adapt to changing environments. The deletion of different two component systems, such as *gacA*/*gacS* (Tan et al., 1999b; Feinbaum et al., 2012; Nandi et al., 2015), *phoP*/*phoQ* (Aballay et al., 2000; Alegado and Tan, 2008; Gellatly et al., 2012), and *kdpD*/*kdpE* (Alegado et al., 2011) resulted in attenuated virulence of the various pathogenic strains. In *Staphylococcus aureus*, the *kdpD/kdpE* two component system has been reported to control different virulence factors, including *spa*, *cap*, *hla*, *aur*, *geh*, and *hlgB* (Zhao et al., 2010). Similarly, in the case of *P. aeruginosa* and *Salmonella typhimurium*, *kdpD* mutants exhibited compromised virulence against *C. elegans* (Alegado et al., 2011; Feinbaum et al., 2012). In another genome-wide mutant library screening, *kdpB* was reported to be required for the full virulence of *P. aeruginosa* PAO1 (Dubern et al., 2015). However, there were no further investigations to explore the possible role of the *kdpD*/*kdpE* two component system and its target operon *kdpFABC* in the pathogenicity of the *Pseudomonas* species. In our qRT-PCR results, the *kdpD* and *kdpB* genes were down regulated by 3.26- and 3.93-fold, respectively, when the worms fed on *P. syringae* MB03 on NGM medium. Previously, similar results were reported in which the *kdpD* gene was down regulated in *Staphylococcus aureus* during phagocytosis by human neutrophils (Voyich et al., 2005). On the other hand, the *kdpD* gene was up regulated during the growth of *Mycobacterium avium* in human macrophages (Hou et al., 2002). A similar response was observed for *kdpD* and *kdpABC* when bacterial cells were exposed to an antibacterial polysaccharide (Mellegard et al., 2011). Taken together, it was hypothesized that mutations in the *kdpD*/*kdpE* two component system might result in attenuated virulence in the *P. syringae* – *C. elegans* infection model. To probe the role of the *kdpD*/*kdpE* two component system in the *P. syringae* – *C. elegans* infection model, the genes were deleted, and the pathogenicity of the mutants was compared with the wild-type strains. It was found that in the *P. syringae* – *C. elegans* model, the *kdpE* mutant showed increased virulence. As far as the *kdpD* mutant is concerned, bacterial virulence was compromised. However, attenuated virulence might be due to retarded growth.

The *phoB/phoR* system has been reported to regulate cellular concentrations of inorganic phosphate (Santos-Beneit, 2015). This system is also reported to play an important role in bacterial pathogenesis in which *phoB* has been reported to directly bind to some virulence factors in *Edwardsiella tarda* (Chakraborty et al., 2011). Similarly, the depletion of external inorganic phosphate has been reported to activate the lethal phenotype of *P. aeruginosa*, which is sensed by the *phoB/phoR* two component system (Zaborin et al., 2009). It was reported that upon the depletion of extracellular phosphate, *phoB/phoR* triggered the production of pyoverdine via the *pqs* quorum sensing system. Interestingly, we observed an up regulation of the *phoB/phoR* and pyoverdine genes during the host–pathogen interaction on PG medium. Importantly, the *pqs* quorum sensing system which made an important link in the mechanism of red death, is absent in *P. syringae* strains (Buell et al., 2003; Feil et al., 2005; Ravindran et al., 2015). In another study on PAO1, *phoB/phoR* mutants showed attenuated virulence, even with normal concentrations of extracellular phosphate (Dubern et al., 2015). Taken together, it can be suggested that *phoB/phoR* is not only involved in the mechanism of red death, but it also seems to regulate other virulence mechanisms.

On the other hand, the role of pyoverdine has been well reported in the killing of *C. elegans* (Zaborin et al., 2009; Kirienko et al., 2013). It was previously reported that in a liquid killing assay, pyoverdine alone is sufficient to kill *C. elegans* (Kirienko et al., 2013). However, the attenuated virulence of pyoverdine mutants was only found in the liquid killing assay whereas in slow killing assay (agar based), no defect in virulence was observed (Kirienko et al., 2013). In another study, a killing assay was conducted on NGM agar using various concentrations of inorganic phosphate, and pyoverdine was found to be a key virulence factor (Zaborin et al., 2009). Pyoverdine, *phoB* and quorum sensing system together resulted in the red death mechanism of *C. elegans* (Zaborin et al., 2009). In our results, the *pvdE* and *pvdJ* genes showed 3.193 ± 0.31 and 10.145 ± 1.13-fold up regulation, respectively, when the interaction took place on PG medium (**Figure 4**). Pyoverdine not only disturbs the iron homeostasis of the host, which results in host death, but it is also believed to regulate other virulence factors, including exotoxins and endoproteases (Lamont et al., 2002). The up regulation of the pyoverdine genes and the *phoB*/*phoR* two component system showed the importance of these systems in bacterial virulence. However, the exact mechanism by which these systems participate in the killing of worms in the *P. syringae* – *C. elegans* infection model needs further exploration.

Together, the results of the transcriptional response of the selected virulence genes in the presence of *C. elegans* and the gut colonization on PG medium provided insights into the mechanism of the pathogenicity of *P. syringae* MB03 against *C. elegans*. It can be suggested that the different two component systems, such as *kdpD/kdpE, phoB/phoR*, and *phoP/phoQ*, helped the bacterial strain sense and adapt to the harsh environmental conditions of the host. Proteases, such as ClpS, ClpA, and PepP, may also have a role in bacterial colonization by strengthening the pathogen’s defense system. It is not clear whether these proteases also have some toxicity against the host. In the background of the transcriptional response of virulence genes, it is easy to understand why the colonization only occurred on PG medium.

In summary, the current study was the first to investigate the pathogenic behavior of *P. syringae* against *C. elegans*. It was found that the pathogenicity was highly dependent upon the growth medium and the killing of worms required a prolonged interaction between the host and pathogen. Through different types of bioassays and the transcriptional analysis of potential corresponding nematicidal genes, this study provided evidence of the harmful interaction of *P. syringae* with *C. elegans* and also provided insight into its pathogenicity mechanism.

## Author Contributions

MA performed most of the experiments, made most of the data evaluation and drafted the manuscript. YS, LX, HY, and AB participated in partial experiments and interpretation of the data. LL conceived and directed the study and revised the manuscript. All authors read and approved the final manuscript.

## Conflict of Interest Statement

The authors declare that the research was conducted in the absence of any commercial or financial relationships that could be construed as a potential conflict of interest.

## Supplementary Material

FIGURE S1 **Schematic representation of gene knock out**. Upstream and downstream fragments of gene of interest were amplified and ligated by SOE PCR. These ligated fragments were inserted into pDS3.0 vector by *Sac*I restriction enzyme site (steps 1 and 2). Recombinant vectors were transferred into *E. coli* WM3064 and finally to *Pseudomonas syringae* by conjugation. Single cross over mutants were screened by gentamicin^+^ and sucrose^-^ phenotype (step 3). Finally, double cross over mutants were identified by gentamicin^-^ and sucrose^+^ phenotype (wild-type phenotype). Primers were designed from flanking region for PCR and knock out was confirmed by sequencing (step 4).

FIGURE S2 **Expression of GFP in *Pseudomonas syringae* MB03, *Pseudomonas syringae* EK519, and *Pseudomonas syringae* DK519**. Plasmid p519ngfp was transferred to *Pseudomonas syringae* MB03 and its mutants (Δ*kdpE* and Δ*kdpD*) by electroporation. Recombinant strains were grown in LB for overnight, washed thoroughly with ddH_2_O and visualized at 100× magnification under fluorescence microscope.
