# Supplementary figures and images for: The Pathogenicity of Pseudomonas syringae MB03 against Caenorhabditis elegans and the Transcriptional Response of Nematicidal Genes upon Different Nutritional Conditions
Source: Front Microbiol. 2016 May 30;7:805. doi: 10.3389/fmicb.2016.00805 (PMC4884745; doi:10.3389/fmicb.2016.00805)

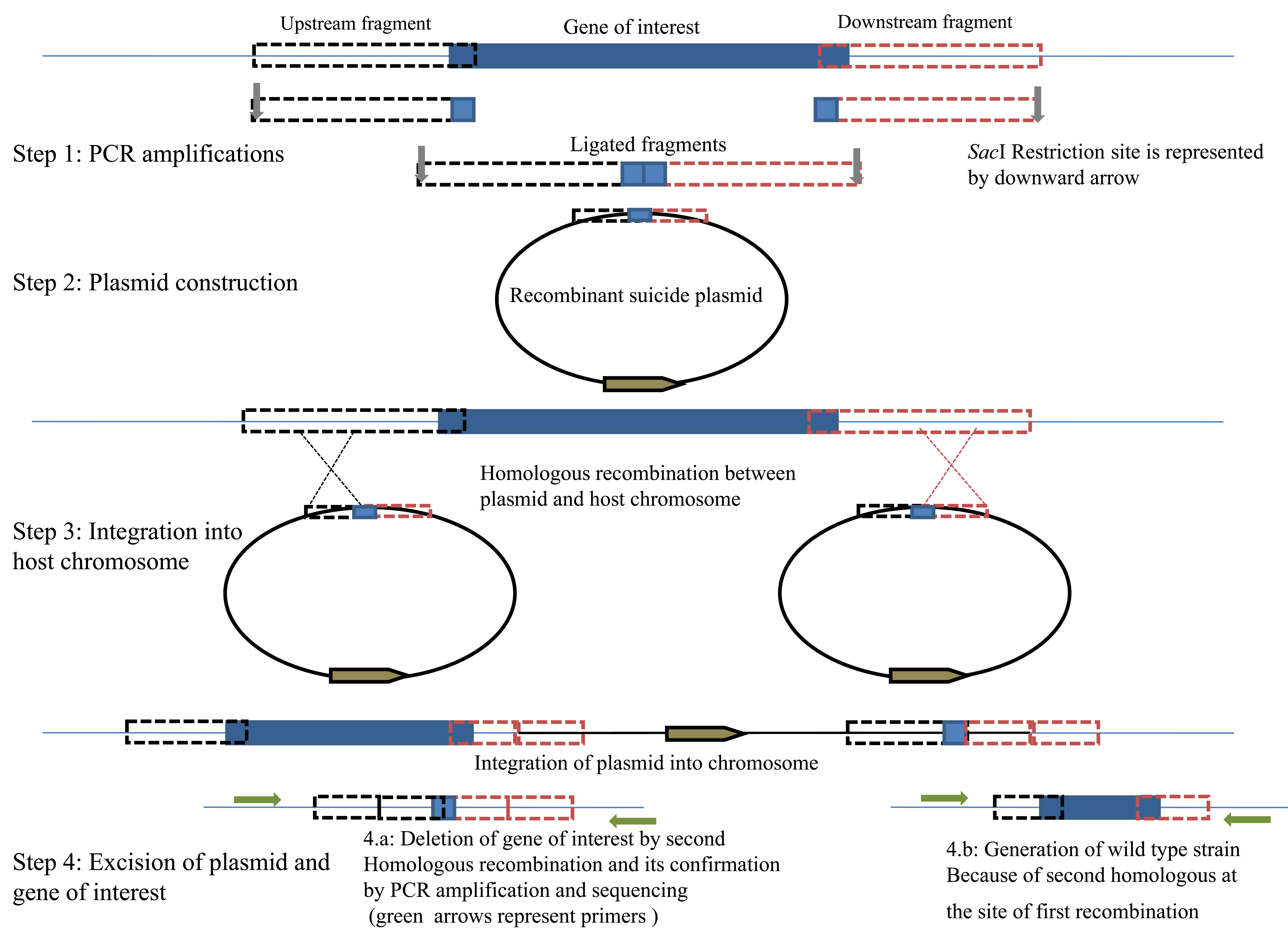

Supplement: FIGURE S1 — Schematic representation of gene knock out. Upstream and downstream fragments of gene of interest were amplified and ligated by SOE PCR. These ligated fragments were inserted into pDS3.0 vector by SacI restriction enzyme site (steps 1 and 2). Recombinant vectors were transferred into E. coli WM3064 and finally to Pseudomonas syringae by conjugation. Single cross over mutants were screened by gentamicin+ and sucrose- phenotype (step 3). Finally, double cross over mutants were identified by gentamicin- and sucrose+ phenotype (wild-type phenotype). Primers were designed from flanking region for PCR and knock out was confirmed by sequencing (step 4). [file Image_1.TIF]

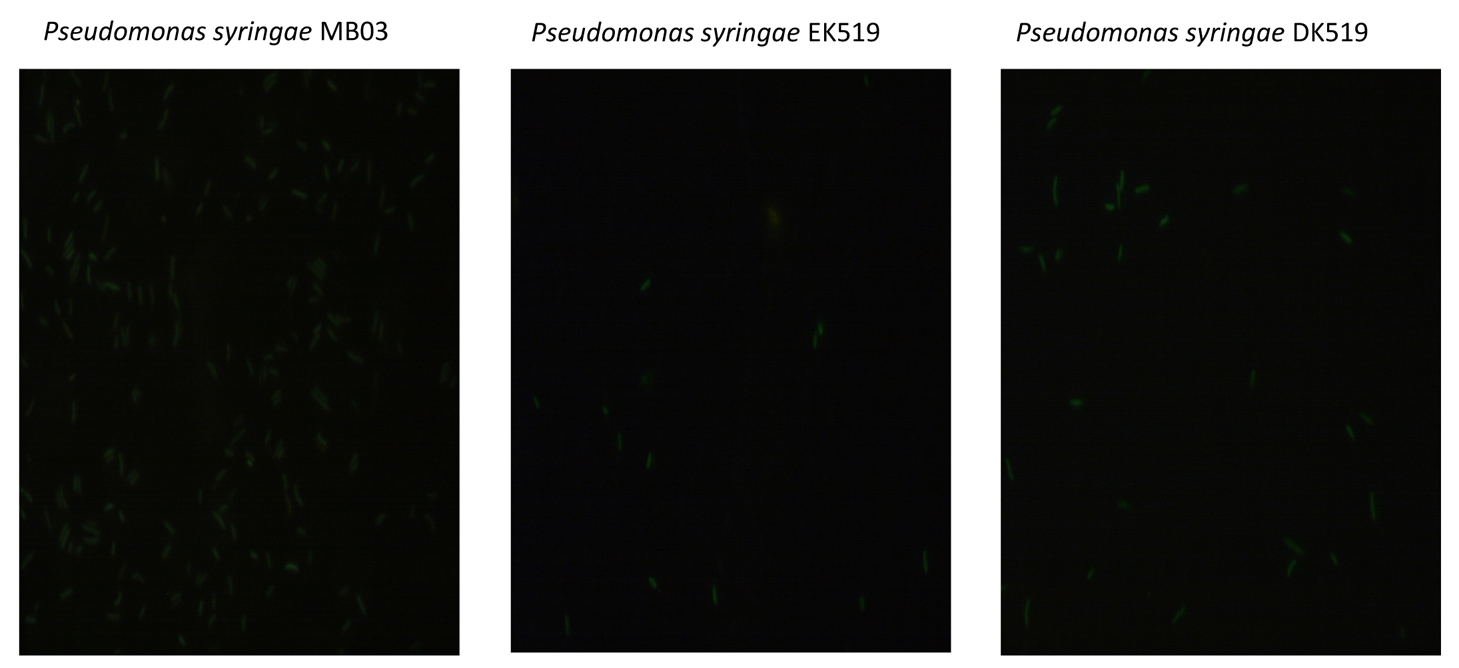

Supplement: FIGURE S2 — Expression of GFP in Pseudomonas syringae MB03, Pseudomonas syringae EK519, and Pseudomonas syringae DK519. Plasmid p519ngfp was transferred to Pseudomonas syringae MB03 and its mutants (ΔkdpE and ΔkdpD) by electroporation. Recombinant strains were grown in LB for overnight, washed thoroughly with ddH2O and visualized at 100× magnification under fluorescence microscope. [file Image_2.TIF]
