# Supplementary material for: The Pathogenicity of Pseudomonas syringae MB03 against Caenorhabditis elegans and the Transcriptional Response of Nematicidal Genes upon Different Nutritional Conditions
Source: Front Microbiol. 2016 May 30;7:805. doi: 10.3389/fmicb.2016.00805 (PMC4884745; doi:10.3389/fmicb.2016.00805)
Supplement: Supplementary file 3 [file Table_1.DOCX]

**Table S1.** Primers used in qPCR and theirs sequences

| Gene | Primer | Primer Sequence |
| --- | --- | --- |
| *16S rRNA gene* | 16S-F | ACACCGCCCGTCACACCA |
|  | 16S-R | GTTCCCCTACGGCTACCTT |
| *algL* | algL-F | CTCAACGCGGTATCCGAAGA |
|  | algL-R | ATCATGTTCAGCGCGCAATC |
| *algU* | algU-F | CCCATGAGGCTCAAGACGTT |
|  | algU-R | CGACCTCGCGAAACCAGATA |
| *aruB* | aruB-F | TACCGAACCGATGCTCGATG |
|  | aruB-R | CAGGTAAGCCCAGACACTCG |
| *clpA* | clpA-F | TTCGGTCGAGGACACCATTG |
|  | clpA-R | GCACCCGCTTCATCAATCAC |
| *clpS* | clpS-F | ACACCCCGATGGATTTCGTC |
|  | clpS-R | GCTCTCCCTGGCGTATTGAT |
| *fleN* | fleN-F | CTTGCCGATGTCATGGAGGG |
|  | fleN-R | GACCACCGACTCACCGATAC |
| *fliD* | fliD-F | TGGTTTCTCTGGTGTCGTCG |
|  | fliD-R | ACTGCGACAGGGAACTCAAG |
| *fliC* | fliC-F | GTTCAGTCGCGAAACGACAG |
|  | fliC-R | GGAGCCAACCTGGAAAGTCA |
| *fimU* | fimU-F | CGATACCGAGCTCAGCGATT |
|  | fimU-R | GTGGGTGTCGCCTGAGTATC |
| *gacA* | gacA-F | CTGGCCGACATTGATGGTCT |
|  | gacA-R | CCTCCGATACCCGGCATTTT |
| *gacS* | gacS-F | ACTCAAGGACGGCAATCTGG |
|  | gacS-R | ATTCTGTCGCACATCCTCGG |
| *hdtS* | hdtS-F | CTGTTGTGGTGCACGTTGAG |
|  | hdtS-R | GCCAGTCACTTCGACCTTGA |
| *kdpD* | kdD-F | AACATCCAGCGCGAAGAAGA |
|  | kdD-R | TGAACACCGCTTTACCGTCA |
| *kdpE* | kdpE-F | ATTTGCTGGTGCTCGACCTT |
|  | kdpE-R | CGGTTTGGTCACGTAGTCGT |
| *kdpB* | kdpB-F | TCTACTGAATCGCAGGCCAC |
|  | kdpB-R | CGATCTGTACGCAGACCGAA |
| *motB* | motB-F | GAAGCGGAAAATGCCGAGAC |
|  | motB-R | CTGGATACGCAAACCGTCCT |
| *motD* | motD-F | CAATTCCCGACCAACTGGGA |
|  | motD-R | GATTGCGCGAGATAACCAGC |
| *mucD* | mucD-F | ATCAAGGAAGTCACAGGCGG |
|  | mucD-R | GAGACAGAGCGATCCTTGGG |
| *minD* | minD-F | AGGAAATCCTTGCCGTCACC |
|  | mind-R | GCACATCCAGAAAACGGTGC |
| *nusA* | nusA-F | TACGAGACTTTCCGTCGCTG |
|  | nusA-R | CAATGACTTGCTTGGCGGTC |
| *pepP* | pepP-F | TGATCTGGTGCTGATCGACG |
|  | pepP-R | TAATCACCTTGACCGTCGCC |
| *phoB* | phoB-F | AAATGATCGCCGTTGCGTTG |
|  | phoB-R | ATGTCCCCGGTCAGTTCATC |
| *phoP* | phoP-F | TTGCTGGACCTCAATCGCAA |
|  | phoP-R | ACCAGCACTTCGATCACGTT |
| *phoQ* | phoQ-F | GCAATGCCGCGTTCAGTATC |
|  | phoQ-R | CACACCGGATTCCACCTGAT |
| *phoR* | phoR-F | GAAAGCAACATGCGCCTCAA |
|  | phoR-R | TTTGGTCTCGATGCCGATCC |
| *pilA* | pilA-F | AGCACATTGGGTAGCTGTGA |
|  | pilA-R | GACTCCAGCCCAAGTGATCT |
| *pilF* | pilF-F | CGCGAGCATTTTACCAAGGC |
|  | pilF-R | GCCGTGTACCGAGCAATAGA |
| *pilO* | pilO-F | TGCGGTCATGATTTTCGCAC |
|  | pilO-R | TCTTGTACGGCTCGAGGTTG |
| *pilR* | pilR-F | GCGCATCGACAAACCTTTCA |
|  | pilR-R | AACAGACCGGGCTTGTCTTC |
| *ptsP* | ptsP-F | GTGAGTGAAGAGCTGACCCC |
|  | ptsP-R | AGACTTCGCCATGATAGCCG |
| *prpB* | prpB-F | CCACGCCATTGTTTACGGTC |
|  | prpB-R | GATCGCGTCATACAGCTCCA |
| *prpC* | prpC-F | CCCGGCGATCATGTGTTACT |
|  | prpC-R | GGCGTACAGAATCAGCGAGA |
| *pvdD* | pvdD-F | GCTTTGCCCGCTATTTCGAC |
|  | pvdD-R | GTTCGACCTGCGAACCAAAC |
| *pvdE* | pvdE-F | GCGACATCGTGTTCATCGTC |
|  | pvdE-R | AACAGCTGGCGGTAGTCATC |
| *pvdJ* | pvdJ-F | GGCGGCCTACGTGATTCATA |
|  | pvdJ-R | AATAGCACGGTATGGCCGAG |
| *pvdL* | pvdL-F | CTGCGCGAGTACGAATACCT |
|  | pvdL-R | AATCCGAGGTGGCATTCAGG |
| *surA* | surA-F | GCCAACATTCTGATCGCCAC |
|  | surA-R | CCGCCTTCCAGTGCATTTTC |
| *recA* | recA-F | GGTCAGATCGAGCGTCAGTT |
|  | recA-R | TTCGGCAGACCACCAATACC |
| *rpoB* | rpoB1-F | TAAGGCACAGTTCGGTGGTC |
|  | rpoB1-R | ACGTTGAAAGACTCGGGCAT |
| *rpoN* | rpoN1-F | GCTCGCAAATCGAATCCACC |
|  | rpoN1-R | GCATGAAGGTGTTGTCTGCG |
| *rpoS* | rpoS1-F | CAATCGTGGGCTGTCATTGC |
|  | rpoS1-R | CGGATGGTCCGTGTCTGATT |
| *Vfr* | vfr-F | ATGCGAAGTGGCCGAGATTT |
|  | vfr-R | TCAAGGAACGCCAGATCACC |
