# Supplementary material for: The Pathogenicity of Pseudomonas syringae MB03 against Caenorhabditis elegans and the Transcriptional Response of Nematicidal Genes upon Different Nutritional Conditions
Source: Front Microbiol. 2016 May 30;7:805. doi: 10.3389/fmicb.2016.00805 (PMC4884745; doi:10.3389/fmicb.2016.00805)
Supplement: Supplementary file 4 [file Table_2.DOCX]

**Table S2. Primers used for markerless gene knockout of *kdpD* and *kdpE* genes**

| Gene | Primer | Sequence | Description |
| --- | --- | --- | --- |
| *kdpD* | DKC-F | ATCGTCGTGCCGTTTATCG | Primers used for the confirmation of *kdpD* knockout. Primers were designed from the region which was not included in gene knockout. |
|  | DKC-R | TGCAGCGGCAGAAACAGAGTG |  |
|  | D5O-F | **ACGAGCTC**GGTCGCACTCGGCCTTGTTTGA | Primers used for the amplification of 5’ flanking region of *kdpD*. Bold red color is for *Sac*I site. Overlapping sequence added for fusion PCR is represented in blue color. Total size of amplified fragment was 1003 bp. |
|  | D5I-R | TCAGTGGCGGCAGTCATCTTGAGCAGCGCGTCGAGGTCCATTT |  |
|  | D3I-F | AAATGGACCTCGACGCGCTGCTCAAGATGACTGCCGCCACTGA | Primers used for the amplification of 3’ flanking region of *kdpD* gene*.*  Bold red color is for *Sac*I site. Overlapping sequence added for fusion PCR is represented in blue color. Total size of amplified fragment was 1584 bp. |
|  | D3O-R | **ACGAGCTC**GATGCCCTCCTCGACCGTTAG |  |
| *kdpE* | EKC-F | TCAGCGCAGCAGAACAGC | Primers were used for confirmation of *kdpE* knockout. Primers were designed from the region which was not included in gene knockout. |
|  | EKC-R | ACGCATTAGCACGATAGTCATTT |  |
|  | E5O-F | **ACGAGCTC**GCCGATCACCGCAGAAGAAG | Primers used for the amplification of 5’ flanking region of *kdpE* gene*.*  Bold red color is for *Sac*I site. Overlapping sequence added for fusion PCR is represented in blue color. Total size of amplified fragment was 1053 bp. |
|  | E5I-R | GAGGATGAACCGCGGGGCGTCGAGCACCAGCAAATCAG |  |
|  | E3I-F | CTGATTTGCTGGTGCTCGACGCCCCGCGGTTCATCCTC | Primers used for the amplification of 5’ flanking region of *kdpE* gene*.*  Bold red color is for *Sac*I site, Overlapping sequence added for fusion PCR is represented in blue color. Total size of amplified fragment was 973 bp. |
|  | E30-R | **ACGAGCTC**GTCCAGACGCCCCATTTTCA |  |
